# Expected clinical utility of automatable prediction models for improving palliative and end-of-life care outcomes: Toward routine decision analysis before implementation

**DOI:** 10.1093/jamia/ocab140

**Published:** 2021-09-02

**Authors:** Ryeyan Taseen, Jean-François Ethier

**Affiliations:** 1 Respiratory Division, Department of Medicine, Faculty of Medicine and Health Sciences, University of Sherbrooke, Sherbrooke, Quebec, Canada; 2 Centre Interdisciplinaire de Recherche en Informatique de la Santé, University of Sherbrooke, Sherbrooke, Quebec, Canada; 3 Groupe de Recherche Interdisciplinaire en Informatique de la Santé, University of Sherbrooke, Sherbrooke, Quebec, Canada; 4 General Internal Medicine Division, Department of Medicine, Faculty of Medicine and Health Sciences, University of Sherbrooke, Sherbrooke, Quebec, Canada

**Keywords:** machine learning, decision support techniques, advance care planning, clinical utility, quality improvement

## Abstract

**Objective:**

The study sought to evaluate the expected clinical utility of automatable prediction models for increasing goals-of-care discussions (GOCDs) among hospitalized patients at the end of life (EOL).

**Materials and Methods:**

We built a decision model from the perspective of clinicians who aim to increase GOCDs at the EOL using an automated alert system. The alternative strategies were 4 prediction models—3 random forest models and the Modified Hospital One-year Mortality Risk model—to generate alerts for patients at a high risk of 1-year mortality. They were trained on admissions from 2011 to 2016 (70 788 patients) and tested with admissions from 2017-2018 (16 490 patients). GOCDs occurring in usual care were measured with code status orders. We calculated the expected risk difference (beneficial outcomes with alerts minus beneficial outcomes without alerts among those at the EOL), the number needed to benefit (number of alerts needed to increase benefit over usual care by 1 outcome), and the net benefit (benefit minus cost) of each strategy.

**Results:**

Models had a C-statistic between 0.79 and 0.86. A code status order occurred during 2599 of 3773 (69%) hospitalizations at the EOL. At a risk threshold corresponding to an alert prevalence of 10%, the expected risk difference ranged from 5.4% to 10.7% and the number needed to benefit ranged from 5.4 to 10.9 alerts. Using revealed preferences, only 2 models improved net benefit over usual care. A random forest model with diagnostic predictors had the highest expected value, including in sensitivity analyses.

**Discussion:**

Prediction models with acceptable predictive validity differed meaningfully in their ability to improve over usual decision making.

**Conclusions:**

An evaluation of clinical utility, such as by using decision curve analysis, is recommended after validating a prediction model because metrics of model predictiveness, such as the C-statistic, are not informative of clinical value.

## INTRODUCTION

End-of-life (EOL) conversations and shared decision making between clinical staff and hospitalized patients can improve the quality of EOL care.^1^^,^^2^ In the hospital setting, these conversations inform goals-of-care (GOC) documentation, particularly code status orders (CSOs), which encode the essential preferences for life-supporting therapy.^3^ Hospitalizations are frequent at the EOL, and in which the need to plan for future care is matched by the opportunity to do so.^4^ However, hospitalized patients with a poor prognosis do not benefit from EOL conversations or GOC documentation as often as they should.^5^ Closing this gap is challenged by workload constraints and difficulty in prognostication.^6^

Clinical decision support systems (CDSSs) that integrate automated prediction models may help increase the prevalence of GOC discussions by generating computerized alerts for patients with a high risk of mortality.^2^^,^^7^ The rationale is that physicians, when explicitly alerted to the poor prognosis of a patient in their care, will initiate a discussion about GOC if one is appropriate and has not already occurred.

In the translational pathway of prediction models, an increasingly recognized step is the assessment of clinical utility,^8^ which should occur before a prospective evaluation of clinical impact.^9^^,^^10^ CDSSs, particularly those with machine learning (ML) models, are potentially costly to implement^11^^,^^12^ and their impact highly subject to local factors,^13^ giving reason to assess clinical value before investing in application. Decision-analytic methods for this assessment using observational data are accessible^10^^,^^14–16^ but are rarely used for prediction models prompting palliative and EOL care (PEOLC) interventions, resulting in poor evidence of value.^17^^,^^18^ A few decision analyses in this area of research have assessed system-perspective monetary value^19^^,^^20^; a decision-analytic evaluation of clinical benefits and harms from the perspective of patient-centered quality improvement has remained elusive.

In this study, we evaluated the clinical utility of locally applicable prediction models using a routinely collected measure of GOC discussions, CSOs in the electronic health record (EHR). Our primary objective was to compare the expected clinical value of a novel ML model with that of a published model^21^ and models requiring fewer types of predictors. In the process, we demonstrate innovative strategies to increase the applicability of simple decision-analytic techniques for assessing the utility of automatable prediction models before implementation.

## MATERIALS AND METHODS

This retrospective study comparing prediction models includes methods for the development, validation, and decision-analytic evaluation of prediction models. We conform to the TRIPOD (Transparent Reporting of a Multivariable Prediction Model for Individual Prognosis or Diagnosis) guidelines^22^ for reporting prognostic modelling methods, and to the relevant aspects of the CHEERS (Consolidated Health Economic Evaluation Reporting Standards) guidelines^23^ for reporting decision-analytic methods. The study took place at an integrated university hospital network with 2 sites and about 700 acute care beds in the city of Sherbrooke, Quebec, Canada (details in the Supplementary Appendix). Institutional Review Board approval was obtained prior to data collection (Institutional Review Board of the CIUSSS de l’Estrie—CHUS #2018-2478 ).

### Source of data and participants

All predictor data in the study was collected from the institutional data warehouse, which combines EHR and administrative data. All adult hospitalizations admitted to a nonpsychiatric service between July 1, 2011, and June 30, 2018, were included in the overall cohort, except for admissions to rarely admitting specialties (eg, genetics) or admissions with a legal context (eg, court ordered). Mortality records were sourced from the Quebec vital statistics registry and considered complete until June 30, 2019 (additional details in the Supplementary Appendix).

The overall cohort was split temporally, with a training cohort defined as admissions occurring between July 1, 2011, and June 30, 2016, inclusively, and a testing cohort defined as hospital admissions occurring between July 1, 2017, and June 30, 2018, inclusively. This split was designed to simulate the prospective evaluation of a given model had it been trained with all available data just before midnight on June 30, 2017, and then applied prospectively for 1 year at our institution. Hospitalizations that occurred between July 1, 2016, and June 30, 2017, inclusively, were excluded to prevent any unrealistic leakage of outcome information between the training and testing cohort.

For the evaluation of clinical utility, our population of interest was all hospitalizations in which there was enough time for a GOC discussion to occur and in which it was not inappropriate or unnecessary given the information available to a CDSS at the point of care. We defined a CDSS-eligible cohort by excluding hospitalizations from the testing cohort that did not have overnight stay or that were admissions in obstetrics or palliative care.

### Prediction models

We developed a ML model using the random forest (RF) algorithm that includes administrative, demographic, and diagnostic predictors accessible at the time of hospital admission to predict 1-year mortality (RF-AdminDemoDx, 244 predictors). As an alternative strategy, we updated the Modified Hospital One-year Mortality Risk (mHOMR) model^21^ for local application (9 predictors). In addition, we specified 2 simplified versions of the RF-AdminDemoDx model: one in which no diagnostic variables were included (RF-AdminDemo, 12 predictors) and one in which only 4 variables—age, sex, admission service, and admission type—were included (RF-Minimal). All 4 prediction models were feasible to operationalize with the existing informatics infrastructure, though had different requirements in terms of data access and implementation (Table 1). Data generation processes were investigated to align retrospectively extracted variables with what would be available within a few minutes of hospital admission. The models were trained with the training cohort, their temporal validity evaluated with the testing cohort, and their clinical utility evaluated with the CDSS-eligible cohort. Model development, specification, and validation is fully described in the Supplementary Appendix.

**Table 1.** Predictors included in automatable prediction models

Variable^a^	Type	Description
Age^b^^,^^c^^,^^d^	Integer	Age at admission in full years since birth
ED visits^b^^,^^d^	Integer	Visits to the emergency department in the year before admission
Ambulance admissions^b^^,^^d^	Integer	Admissions to the hospital by ambulance in the year before admission
Weeks recently hospitalized^b^	Integer	Full weeks hospitalized in the 90 d before admission
Sex^b^^,^^c^^,^^d^	Categorical	Female or male
Living status^b^^,^^d^	Categorical	Chronic care hospital, nursing home, home, or unknown^e^
Admission type^b^^,^^c^	Categorical	Urgent, semi-urgent, elective, or obstetric
Admission service^b^^,^^c^^,^^d^	Categorical	Cardiac surgery, cardiology, critical care, endocrinology, family medicine, gastroenterology, general surgery, gynecology, hematology-oncology, internal medicine, maxillofacial surgery, nephrology, neurosurgery, neurology, obstetrics, ophthalmology, orthopedic surgery, otorhinolaryngology, palliative care, plastic surgery, respirology, rheumatology, thoracic surgery, trauma, urology, or vascular surgery
Admission diagnosis	Binary set	Free-text diagnosis on admission order form mapped to 147 binary variables using regular expressions (see Supplementary Appendix)
Comorbidity groups	Binary set	ICD-10 codes from hospital discharge abstracts and ED information systems mapped to 84 binary variables (see Supplementary Appendix)
Visible comorbidities	Binary	If a previous hospitalization occurred between 5 y and 6 mo before admission or if a previous ED visit occurred between 6 mo and 2 wk before admission
Flu season^b^^,^^f^	Binary	If the current admission is in the month of December, January, or February
ICU admission^b^^,^^d^	Binary	If the current admission is a direct admission to the ICU
Urgent 30-d readmission^b^^,^^d^	Binary	If the current admission is an urgent readmission within 30 d of a previous discharge
Ambulance admission^b^^,^^d^	Binary	If the current admission is via ambulance
ED admission^d^	Binary	If the current admission is via the ED

See Supplementary Appendix for additional details.

ED: emergency department; ICD-10: International Classification of Diseases–Tenth Revision; ICU: intensive care unit.

a All variables except ED admission included in the RF-AdminDemoDx model.

b Included in the RF-AdminDemo model.

c Included in the RF-Minimal model.

d Included in the Modified Hospital One-year Mortality Risk model. For variable transformations and interaction terms, see original specification by Wegier et al.^21^

e Unknown if no previous hospitalization between 5 years and 6 months before admission.

f Models were developed prior to the COVID-19 (coronavirus disease 2019) pandemic; future revisions will likely exclude this variable.

### Perspective

The evaluation of clinical utility was conducted from the perspective of a clinician-led quality improvement team that aims to implement a CDSS to increase the prevalence of GOC discussions for patients with a poor prognosis: a promising initiative^3^ for a well-established problem.^5^ A necessary component of GOC discussions for hospitalized patients are discussions about code status, the documentation of which had been standardized as CSOs in the institutional EHR since 2015. The documentation of resuscitation preferences for a hospitalized patient with a poor prognosis is a positively valued, patient-centered outcome in the context of EOL communication,^24^ and its absence for the same population is considered a potentially harmful medical error.^2^^,^^25^ The main objective of decision analysis was to identify the prediction model that maximized this quality indicator. The secondary objective was evaluating the net benefit (NB)^14^^,^^15^ of prediction models.

### Alternative strategies

We simulated the operation of a CDSS that uses alternative prediction models for triggering an alert. Conceptually, alerts would suggest discussing GOC, including cardiopulmonary resuscitation (CPR) preferences, if appropriate,^7^ and remind physicians to document CPR preferences in a CSO. The system would generate alerts after midnight for eligible patients admitted the previous day having a predicted risk greater or equal to a certain risk threshold. Because intervention harm was minimal and time constraints were known to limit GOC discussions,^2^^,^^6^ we considered the proportion of admissions with an alert, or alert prevalence, to be the most appropriate criteria for determining risk thresholds. For example, if it was preferable for alert prevalence to be 10%, then we would set the risk threshold of each prediction model to be the 90th percentile of predicted risk: 10% of cases would have a risk higher than the threshold, leading to an alert. We set an alert prevalence of 10% as a point of reference and expected 5% to 20% to be an appropriate range for sensitivity analyses.

The alternative strategies under consideration were the 4 mortality alert rules that resulted from applying to each prediction model an alert prevalence–fixed risk threshold.

### Outcome definitions

Electronic CSOs were linked to hospitalizations in the testing cohort after model development and did not have any role in predictive validation. These orders could convey 1 of 3 resuscitation preferences: wants all resuscitation measures (Full code), does not want CPR but wants endotracheal intubation if necessary (do not resuscitate [DNR]/Intubation-OK), and does not want CPR or intubation (DNR/DNI [do not intubate]). We considered a CSO to have occurred during a hospitalization if at least 1 was documented between 1 week before the admission date and the discharge date, inclusively. The extra week was added to associate a hospitalization with any CSOs documented during observation in the emergency department prior to hospital admission. The main outcome was a hospitalization with a CSO among those for patients at the EOL, which we defined as death within 1 year of admission.

### Decision trees

**Figure 1.**
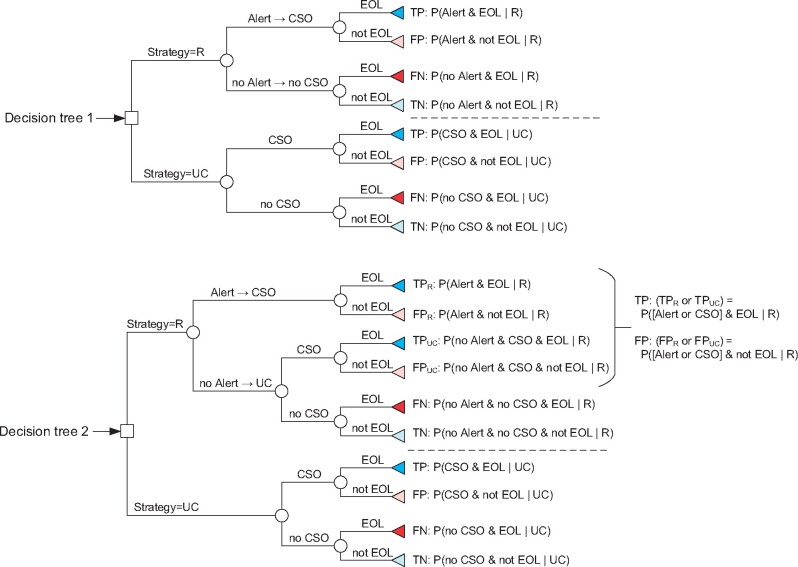
Strategic decision tree models. Two decision trees modelling the potential outcomes of each hospitalization in the clinical decision support system (CDSS)–eligible cohort under alternative strategies. The strategy of an alert rule, R, implies that a CDSS is implemented and uses R to generate alerts. In the strategy of usual care (UC), code status orders (CSOs) occur as they factually did between July 2017 and July 2018 in the 2 hospitals of Sherbrooke, Quebec, Canada. In both trees, an alert always implies a CSO (Alert → CSO; the arrow notation expresses a strategy: if Alert, then do CSO). The difference between the 2 trees is how outcomes unfold in the absence of an alert. In decision tree 1, no alert results in no action (no Alert → no CSO: if no alert, then do no CSO). In decision tree 2, no alert results in the action that occurred retrospectively in usual care (no Alert → UC: if no alert, then do usual care). The first tree models the conventional scenario of decision curve analysis in which a prediction rule aims to reduce intervention-related harm, while the second models the scenario of a CDSS that aims to increase a routine good practice that is constrained by time. A true positive (TP) outcome occurs when a CSO is documented during a hospitalization for a patient who died within 1 year of admission (end-of-life [EOL] status). A false positive (FP) outcome occurs when a CSO is documented during a hospitalization for a patient who survives more than a year (“not EOL” status). A false negative (FN) outcome occurs when no CSO is documented during an EOL hospitalization. A true negative (TN) occurs when no CSO is documented for a “not EOL” hospitalization. The formulas to calculate the expected probability of each outcome for a given strategy are provided to the right of each terminal node.

We modeled 2 decision trees in which alerts led to the desired action (Figure 1). The first was based on the conventional assumptions of decision analysis for prediction models,^26^^,^^27^ in which alerts lead to action and the absence of an alert leads to inaction. The second was a scenario-appropriate adaptation that allowed assessing expected utility relative to a strategy of usual care, in which alerts lead to action and the absence of an alert leads to usual care: either action or inaction depending on what had factually occurred for the alert-negative case. For both trees, the benefit to patients of discussing and documenting GOC^2^ was attributed to true positives (TPs). The cost of this action is spending clinical time,^2^ which was attributed to false positives (FPs). Our valuation procedure and assumptions are further explained in the Supplementary Appendix.

To distinguish the effect of model-based predictions from the effect of simply generating alerts, we included a fifth model of uniformly random numbers between 0 and 1. We did not expect alerts from such a model to cause physicians to act in the same way as the validated prediction models, but it would serve to make explicit a side effect of the assumption that all alerts would cause the desired action of a CSO.

### Statistical analysis

We described cohort characteristics stratified by EOL and CSO status. We assessed model discrimination using the C-statistic and its calibration using a calibration plot.^8^ To assess construct validity of predictions, we regressed DNR preference against predicted risk in the CDSS-eligible cohort.

Our primary measure of expected clinical utility was the expected risk difference (eRD) compared with usual care of the main outcome, calculated for each rule as (1)eRD=P(Alert or CSO | EOL)-P(CSO | EOL)

This metric is based on the intention-to-treat estimator^10^ and answers the hypothetical question: if every alert based on rule *R* had led to the desired action (a CSO), how many more hospitalizations at the EOL (as a proportion of all hospitalizations at the EOL) would have had a CSO?

For contextualizing the eRD, we calculated the number needed to benefit (NNB): (2) NNB=P(Alert)PAlert & EOL-P(Alert & EOL & CSO)

The NNB is the number of alerts needed to increase benefit by one outcome over usual care, assuming that every alert leads to action. It is the reciprocal of the difference in risk of benefiting with a model-based strategy minus the risk of benefiting with usual care (among those identified by an alert): P(*EOL* | *Alert*) – P(*EOL* & *CSO* | *Alert*). Conceptually, it incorporates both the number needed to screen, 1/P(*EOL* | *Alert*), and the number needed to treat, 1/P(*no CSO* | *EOL*), as originally described,^16^ but it was calculated without assuming conditional independence: the subset of patients at the EOL successfully screened with an alert would not necessarily have the same chance of beneficial treatment (the counterfactual outcome in the event of “no CSO”) as the set of all patients at the EOL.

Our secondary measure of expected clinical utility was the NB, calculated for each strategy, *S*, as (3) NBS=PTP S)-PFP S)×Exchange Rate

In our scenario, the risk threshold was based on estimated availability of clinical time, not necessarily patient-provider preference for CSO. This made the risk threshold potentially unsuitable to inform the exchange rate, calculated in conventional decision curve analysis as *Risk Threshold*/(1-*Risk Threshold*).^15^^,^^27^ The exchange rate represents the theoretical ratio between the harm of inappropriate inaction (false negative [FN]) and the harm of inappropriate action (FP), which can be obtained by various means^14^; the threshold method is a convenient simplification in the absence of other utility estimates in the validation set.^27^ We calculated a model-independent exchange rate using observed actions of clinicians (ie, using their revealed preferences)^14^ who implicitly decide under uncertainty between the harm of inaction and the time cost of action: (4) Observed Exchange Rate=P(TP | Usual care)P(FP | Usual care)

Substituting equation 4 in equation 3 results in a NB of zero for the default strategy S = Usual care. We plotted decision curves for both decision trees and for both a threshold-based and observed exchange rate. A visual guide to interpreting the eRD, NNB, and observed exchange rate is provided in the Supplementary Appendix (Supplementary Figure S1 ).

We performed a 2-way sensitivity analysis^14^ between alert prevalence and the exchange rate in subgroups of service type and hospital site. We assessed subgroup heterogeneity using a forest plot of the expected relative risk (the ratio of the terms in the eRD) in relevant inpatient populations.

Consistent with the decision-analytic design, no *P*-valued significance tests were performed between the alternative strategies.^28^ We bootstrapped 95% confidence intervals (CIs) for estimates of predictive accuracy.^8^^,^^29^ To verify the potential influence of including all hospitalizations in the CDSS-eligible cohort, rather than sampling unique patients, we repeated analyses using the first, last, and a random hospitalization per patient. All statistical analyses were performed with R version 3.6.3 (R Foundation for Statistical Computing, Vienna, Austria) (relevant extensions and details in the Supplementary Appendix).^30^

## RESULTS

### Sample and model description

**Figure 2.**
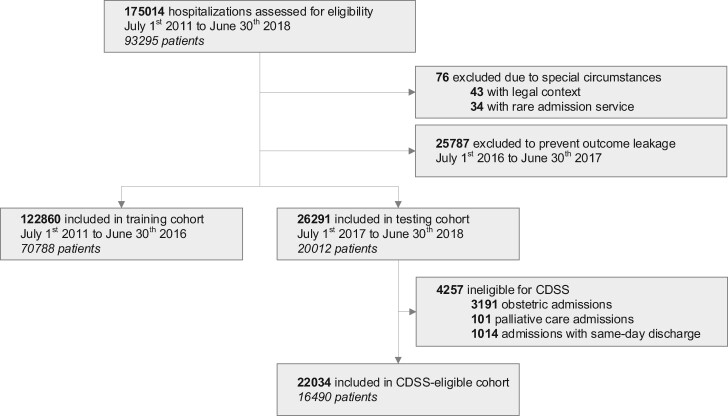
Participant flow diagram. CDSS: clinical decision support system.

**Figure 3.**
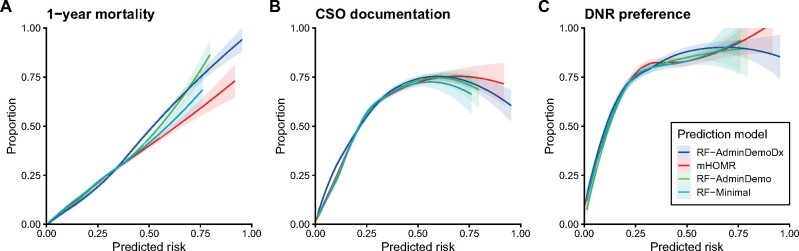
Regression of end-of-life outcome and communication indicators against model-predicted risk. Binary variables regressed against predicted risk of a given model then plotted along with 95% confidence interval bands using the LOESS algorithm. One random hospitalization per patient sampled from the clinical decision support system–eligible cohort before applying regression. (A, B) Showing 16 490 patients, in which 2248 died within 1 year of hospitalization and 5241 had code status order (CSO) documentation during that hospitalization. (C) Showing 5241 patient hospitalizations with CSO documentation, in which 3552 preferred a do not resuscitate (DNR) status in the last CSO documented before discharge. Among the 5241 patients with a CSO, a predicted risk of mortality exceeding 10% to 15% was associated with a majority preference for DNR—either “DNR/Intubation-OK,” or “DNR/DNI (do not resuscitate).” mHOMR: Modified Hospital One-year Mortality Risk.

The participant flow diagram is presented in Figure 2. Between July 1, 2011, and June 30, 2018, there were 175 041 hospitalizations for adults in a nonpsychiatric service at our institution (93 295 patients). After excluding 76 hospitalizations with rare circumstances, the training cohort included 122 860 hospitalizations between July 1, 2011, and June 30, 2016 (70 788 patients), and the testing cohort included 26 291 hospitalizations between July 1, 2017, and June 30, 2018 (20 012 patients). There were 22 034 hospitalizations (16 490 patients) in the CDSS-eligible cohort. Patient-hospitalization characteristics are presented for the CDSS-eligible cohort in Table 2 (description of other cohorts in the Supplementary Appendix). Prediction models had acceptable temporal validity (Table 3; Supplementary Figure S2). When sampling over unique patients, the C-statistic ranged from 0.84 to 0.89 in the testing cohort and lowered to 0.79 to 0.86 in the CDSS-eligible cohort. Figure 3 describes EOL process indicators as a function of model-predicted risk; all models had good construct validity for DNR preferences.

**Table 2.** CDSS-eligible cohort characteristics

	Overall (N = 22 034)	With CSO (n = 7648)	Hospitalizations at EOL (n = 3773)	
			With CSO (n = 2599)	Without CSO (n = 1174)
Age, y	68 (57-78)	77 (67-85)	78 (69-87)	69 (60-78)
Female	10 473 (48)	3817 (50)	1212 (47)	509 (43)
Hospital site				
A	13 350 (61)	3867 (51)	1455 (56)	909 (77)
B	8684 (39)	3781 (49)	1144 (44)	265 (23)
Service type				
Medical	12 187 (55)	6246 (82)	2135 (82)	658 (56)
Surgical	9266 (42)	1023 (13)	312 (12)	484 (41)
Critical care^a^	581 (3)	379 (5)	152 (6)	32 (3)
Admission type				
Nonelective	16 780 (76)	7298 (95)	2525 (97)	917 (78)
Elective	5254 (24)	350 (5)	74 (3)	257 (22)
Living status at discharge^b^				
Home	11 205 (51)	2663 (35)	467 (18)	507 (43)
Home with health center (CLSC) liaison	6115 (28)	1656 (22)	482 (19)	404 (34)
Short-term transitional care	1354 (6)	717 (9)	201 (8)	99 (8)
Nursing home	1612 (7)	1143 (15)	296 (11)	57 (5)
Chronic care hospital	528 (2)	454 (6)	200 (8)	24 (2)
Other^c^	291 (1)	151 (2)	89 (3)	18 (2)
Death in hospital	929 (4)	864 (11)	864 (33)	65 (6)
ED visits^d^				
0	12 786 (58)	3 457 (45)	956 (37)	608 (52)
1-2	6476 (29)	2620 (34)	941 (36)	383 (33)
3 or more	2772 (13)	1571 (21)	702 (27)	183 (16)
Admissions by ambulance^d^				
0	18 547 (84)	5432 (71)	1644 (63)	964 (82)
1-2	2891 (13)	1760 (23)	738 (28)	180 (15)
3 or more	596 (3)	456 (6)	217 (8)	30 (3)
Weeks recently hospitalized^e^				
0	18 601 (84)	5925 (77)	1774 (68)	815 (69)
1-2	2617 (12)	1237 (16)	571 (22)	281 (24)
3 or more	816 (4)	486 (6)	254 (10)	78 (7)
ED admission	12 711 (58)	6329 (83)	2155 (83)	566 (48)
Ambulance admission	7418 (34)	4536 (59)	1628 (63)	297 (25)
Urgent 30-d readmission	2396 (11)	1205 (16)	596 (23)	210 (18)
ICU admission	1036 (5)	454 (6)	171 (7)	51 (4)
ICU stay during hospitalization	3512 (16)	1616 (21)	538 (21)	152 (13)
Hospital length of stay, ^d^	4 (2-8)	7 (4-15)	9 (4-17)	4 (2-8)
Code status preference^f^				
Full code	2323 (11)	2323 (30)	285 (11)	0 (0)
DNR/Intubation-OK	928 (4)	928 (12)	254 (10)	0 (0)
DNR/DNI	4397 (20)	4397 (57)	2060 (79)	0 (0)
Not documented	14 386 (65)	0 (0)	0 (0)	1174 (100)
Major comorbidities^g^				
Congestive heart failure	2758 (13)	1728 (23)	745 (29)	194 (17)
Chronic pulmonary disease	4626 (21)	2516 (33)	922 (35)	265 (23)
Dementia	1815 (8)	1447 (19)	570 (22)	77 (7)
Metastatic cancer	1787 (8)	848 (11)	634 (24)	304 (26)

Values are n (%) or median (interquartile range). Percentages may not add to 100 due to rounding.

CDSS: clinical decision support system; CLSC: Centre local de services communautaires; CSO: code status order; DNI: do not intubate; DNR: do not resuscitate; ED: emergency department; EOL: end of life; ICU: intensive care unit.

a Represent direct admissions to the ICU before a primary non–critical care service could be specified (ie, the responsible service upon ICU discharge). ICU exposure is more precisely measured with the variables “ICU admission” and “ICU stay during hospitalization.”

b See Supplementary Appendix for characteristics of real-time–accessible living status (used for prediction models).

c Includes transfer to another hospital, rehabilitation center, palliative care center, or discharge against medical advice.

d In the year before admission.

e In the 90 days before admission.

f Last preference documented during hospitalization if one was documented.

g Charlson comorbidities using International Classification of Diseases–Tenth Revision codes by Quan et al^31^ and ascertained using the discharge abstract of index hospitalization and of those in the year before discharge.

**Table 3.** Predictive performance of automatable prediction models for the outcome of 1-year mortality

	RF-AdminDemoDx	RF-AdminDemo	RF-Minimal	mHOMR
Internal validation^a^				
C-statistic (range)	0.90 (0.90-0.91)	0.86 (0.85-0.87)	0.85 (0.84-0.86)	0.86 (0.85-0.86)
Brier score (range)	0.068 (0.065-0.073)	0.079 (0.077-0.083)	0.082 (0.078-0.084)	0.081 (0.078-0.085)
External validation^b^^,^^c^				
C-statistic (95% CI)	0.89 (0.88-0.89)	0.85 (0.84-0.86)	0.84 (0.83-0.84)	0.84 (0.83-0.85)
Brier score (95% CI)	0.074 (0.072-0.076)	0.084 (0.081-0.086)	0.086 (0.084-0.089)	0.086 (0.083-0.088)
CDSS-eligible validation^b^^,^^d^				
C-statistic (95% CI)	0.86 (0.85-0.87)	0.81 (0.80-0.82)	0.79 (0.78-0.80)	0.80 (0.79-0.81)
Brier score (95% CI)	0.088 (0.085-0.091)	0.10 (0.097-0.10)	0.10 (0.10-0.11)	0.10 (0.099-0.11)

CDSS: clinical decision support system; CI: confidence interval; mHOMR: Modified Hospital One-year Mortality Risk.

a Internal validity estimated using 10-fold cross-validation in the training cohort (12 069-12 521 hospitalizations and 7078-7079 patients in each fold). Metrics calculated for each fold after sampling 1 random hospitalization per patient. Data given as median estimate (range [ie, minimum-maximum]) across the 10 folds.

b Metrics calculated on 1000 two-stage bootstrapped samples as detailed in the Supplementary Appendix. Data given as median estimate (95% CI).

c Temporal validity estimated in testing cohort (26 291 hospitalizations, 20 012 patients).

d Temporal validity estimated in CDSS-eligible cohort (22 034 hospitalizations, 16 490 patients).

### CSOs at the EOL

There were 7648 hospitalizations associated with a CSO in the CDSS-eligible cohort (35%). Among these, 2599 (34%) were associated with death within 1 year of admission; clinicians were observed to document a CSO during 1 hospitalization at the EOL for every ∼1.9 hospitalizations not at the EOL (observed exchange rate of 2599 TPs to 5049 FPs). On average, clinicians acted as though the harm of FNs was 1.9 times as harmful as a FP.

There were 3773 (17%) hospitalizations at the EOL in the CDSS-eligible cohort. Among these, a CSO was not documented in 1174 cases, meaning a minimal GOC discussion had not been documented for 31% of applicable hospitalizations with overnight stays at the EOL. Compared with hospitalizations at the EOL that did have a CSO, these cases were more likely to be elective (odds ratio, 9.6; 95% CI, 7.3 to 12.5), in surgical specialties (odds ratio, 5.1; 95% CI, 4.4 to 6.1), for younger patients (mean age 68.3 years vs 76.9 years; 95% CI, -9.5 to -7.6]), and of shorter duration (mean length of stay 6.5 days vs 12.9 days; 95% CI, -7.1 to -5.7]).

### Expected clinical utility

**Figure 4.**
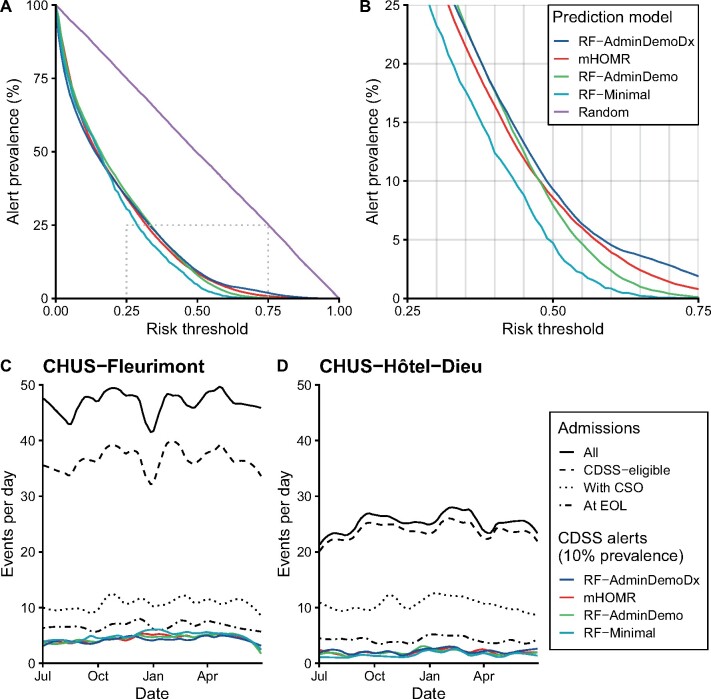
Association between risk threshold and alert prevalence. (A) Alert prevalence is plotted as a function of risk threshold in the clinical decision support system (CDSS)–eligible cohort; Alert prevalence = f(Risk Threshold) = P(Predicted risk ≥ Risk threshold). A region of interest is outlined in which risk thresholds satisfy reasonable workload demands (Alert prevalence of 5-20%). (B) The region of interest in panel A is magnified. (C, D) Time series between July 1, 2017, and June 30, 2018, of daily events stratified by hospital site (CHUS-Fleurimont refers to site A and CHUS-Hôtel-Dieu refers to site B in the main text). The frequency of actual admissions is compared with the frequency of simulated alerts at risk thresholds corresponding to an overall alert prevalence of 10%. “All” refers to all admissions in the testing cohort (excluding pediatric and psychiatric admissions). The loess algorithm was used to smooth day-to-day variations using a span of 0.2 for all curves. Non–CDSS-eligible cases at site A include mostly obstetrical admissions. CHUS: Centre Hospitalier Universitaire de Sherbrooke; CSO: code status order; EOL: end of life; mHOMR: Modified Hospital One-year Mortality Risk.

**Figure 5.**
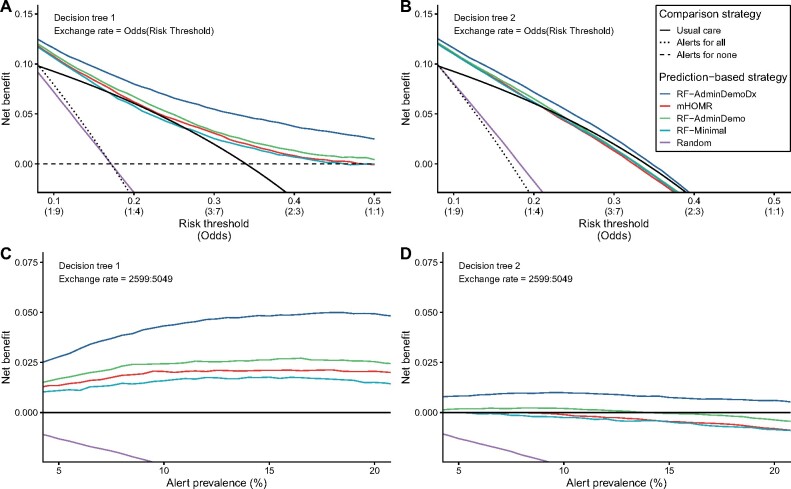
Decision curve analysis. Decision curves to assess net benefit as a function of either desired risk threshold (top) or desired alert prevalence (bottom), and using either decision tree 1 (left) or decision tree 2 (right). In all scenarios, the RF-AdminDemoDx model had a higher net benefit than alternative prediction models. (A) The decision curves use the original methodology of Vickers et al.^27^^,^^32^ (B) Decision tree 2 is assumed instead of the original tree. (C) The exchange rate is assumed to equal the observed exchange rate, rather than the odds at the risk threshold. (D) Combination of the assumptions in panels B and C: decision tree 2 is assumed and the exchange rate is assumed to equal the observed exchange rate. Only the RF-AdminDemoDx and RF-AdminDemo model exceed the net benefit of usual care under these combined assumptions. With decision tree 1, “Alerts for none” and “Alerts for all” implies “CSO for none” and “CSO for all,” respectively. With decision tree 2, “Alerts for all” still implies “CSO for all,” but “Alerts for none” implies “Usual care.” The strategy of “Alerts for all” is a distant outlier in the bottom panels, corresponding to a constant net benefit of around -0.25. The strategy of “Alerts for none” overlaps “Usual care” in panels B to D. CSO: code status order; mHOMR: Modified Hospital One-year Mortality Risk.

Simulated at an alert prevalence of 10%, each model would have generated on average 6 alerts per day over 1 year (Figure 4). At this same level of resource use, the eRD varied between 5.4% and 10.7%, and the NNB between 5.4 and 10.9 alerts (Table 4). The RF-AdminDemoDx model had the highest expected benefit by either metric. This model also maximized NB in the decision curves regardless of the decision tree or exchange rate used (Figure 5). When routine clinical actions were considered, only the RF-AdminDemoDx and RF-AdminDemo models could increase value above usual care in the range of reasonable alert prevalence (Figure 5D).

**Table 4.** Expected clinical utility of prediction models in the CDSS-eligible cohort

	RF-AdminDemoDx	mHOMR	RF-AdminDemo	RF-minimal
Parameters				
Alert prevalence, % (no. alerts)	10 (2204)	10 (2204)	10 (2204)	10 (2205)
Risk threshold	0.478	0.461	0.465	0.422
Clinical utility				
eRD, %	10.7	5.5	6.9	5.4
NNB, alerts	5.4	10.7	8.4	10.9
Benefit	0.1363	0.1273	0.1298	0.1272
Harm	0.1264	0.1284	0.1278	0.1297
Net benefit	0.0099	−0.0011	0.0020	−0.0025
Predictive accuracy				
PPV, %	63	48	50	44
NPV, %	88	86	87	86
Sensitivity, %	37	28	29	26
Specificity, %	95	94	94	93
C-statistic^a^	0.85	0.79	0.80	0.77
Brier score^a^	0.11	0.12	0.12	0.12

Risk threshold set as the 90th percentile of predicted risk in the sample, which results in an alert prevalence of 10%. Sample size = 22 034 hospitalizations; true positive code status orders in usual care = 2599; false positive code status orders in usual care = 5049; observed exchange rate = 0.515. The expected clinical utility of completely random alerts provided for reference in the Supplementary Appendix (Supplementary Table S6).

CDSS: clinical decision support system; eRD: expected risk difference; mHOMR: Modified Hospital One-year Mortality Risk; NNB: number needed to benefit; NPV: negative predictive value; PPV: positive predictive value.

a Threshold independent. These metrics differ for the CDSS-eligible cohort in Table 3 due to the different sampling unit (1 hospitalization per patient is sampled for the results of Table 3; all hospitalizations are included here).

### Sensitivity analysis

**Figure 6.**
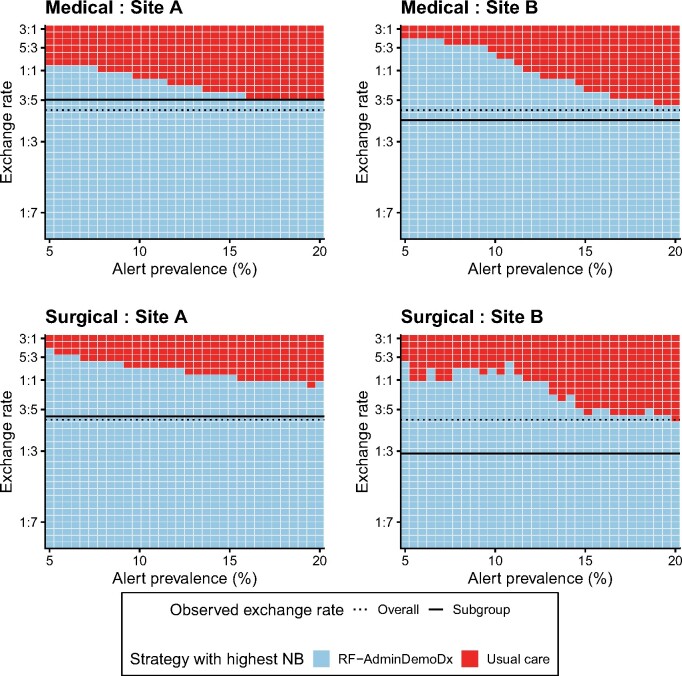
Two-way sensitivity analysis between resource availability and clinical preference. The net benefit (NB) was calculated for each strategy, for each tile, and for each plot; the strategy with the highest NB is indicated for the corresponding combination of alert prevalence and exchange rate in each hospital site (column) and service type (row). For example, if an alert prevalence around 15% was desirable for medical services at site B, and the cost of a false negative (FN) in this group was considered equal to the cost of a false positive (FP) (exchange rate 1:1), then the most beneficial strategy would be usual care. In the same setting, the RF-AdminDemoDx model would be a better strategy if either a lower alert prevalence was acceptable (eg, around 11%, implying a higher risk threshold), or if the cost of FP alerts were relatively lower (eg, exchange rate of 3:5, in which 3 FNs are as costly as 5 FPs). A higher exchange rate indicates a greater preference for avoiding the time cost of a goals-of-care discussion when one is unnecessary (worried about FPs), while a lower exchange rate indicates a greater preference for avoiding the harm of omitting a goals-of-care discussion when one is necessary (worried about FNs). The overall exchange rate (dotted line) was calculated using equation 4 in the full cohort, and the subgroup exchange rate (solid line) corresponds to the result of equation 4 among a given subgroup. The Modified Hospital One-year Mortality Risk, RF-AdminDemo, and RF-Minimal models are not referenced because they were never a strategy with the highest net benefit.

The net benefit of the RF-AdminDemoDx model remained the highest among models in the 2-way sensitivity analysis (Figure 6). Subgroup analysis indicated heterogeneity that could influence implementation, including a smaller benefit for all models at site B (Supplementary Figures S3-S7). Estimates of clinical utility using different sampling strategies did not change the direction or interpretation of results (Supplementary Tables S7-S9, Supplementary Figures S8-S10).

## DISCUSSION

Improving patient identification for routine PEOLC interventions is a priority for healthcare stakeholders aiming to reconcile the default policies of life-sustaining therapy with the static truth that all life comes to an end. We performed an up-to-date review of model validation studies in this area of research and provide both a narrative and tabular synthesis of related studies in the Supplementary Appendix. In recent years, there has been a shift from manual screening tools^33^ toward automated trigger tools,^34^ with the latter shifting from query-based algorithms^35^ toward increasingly flexible, but infrastructure-dependent, prediction models.^21^^,^^36–39^ A challenge with such models is that their usual learning objective, minimizing the error of mortality prediction, is only indirectly related to the clinical objective of maximizing benefit for a resource-limited PEOLC intervention. In this setting of mismatched expectations, predictiveness does not mean usefulness, making it essential to assess clinical utility and not just predictive accuracy.^8–10^^,^^16^

While the most accurate model ended up having the highest expected value, relying on an association between predictiveness and value can be misleading. In Table 4, the RF-AdminDemo and mHOMR model have similar indicators of accuracy (eg, Brier score 0.12 for both; C-statistic 0.80 vs 0.79), but only one would be more beneficial than usual care under the assumptions of decision analysis (net benefit above zero vs net benefit below zero). The unreliability of the C-statistic to discriminate value is even more apparent when comparing a given model’s expected benefit across the 2 hospital sites (Supplementary Figures S3-S6). For the RF-AdminDemoDx model, the C-statistic at both site A and site B was 0.85. The NNB, however, was 4.4 and 11.0, respectively, reflecting a greater usual tendency to document CSOs at site B. The difference between sites was most dramatic for the RF-Minimal model, which had an NNB of 8.8 vs 114: only 4 alerts of 456 simulated during 1 year at site B identified an individual at the EOL that did not already have a CSO documented. The C-statistic was entirely uninformative of this difference, being 0.77 at both sites.

In our review of the literature, we did not find any retrospective study evaluating the clinical utility—both benefits and harms—of automatable prediction models for prompting PEOLC interventions. In contrast, almost all studies reported the C-statistic for mortality, and these were generally above 0.8. The context insensitivity of the C-statistic makes it practical for research but uninformative for practice: more value-based metrics are required to guide decision makers.^14^^,^^17^ Prediction models for prompting a PEOLC intervention had varying use cases for decision support, including GOC discussion, palliative care referral, outpatient follow-up for advance care planning, or hospice referral. The benefits, harms, and resources associated with these actions differ between each other and between health systems; one curve does not fit all.

Strengths of our study included ensuring that retrospectively accessed data represented real-time data and the use of temporal rather than random splitting for validation: simulating prospective application at the point of care. When validated in similar cohorts (not necessarily target population), all models in our study had similar C-statistics as published models (ie, above 0.8). However, the eRD and NNB for a patient-centered outcome ranged almost 2-fold, and only 2 models had a higher NB than usual care with our scenario-appropriate decision tree.

Others have validated the predictive performance of a model, then described physician opinion about the appropriateness of high-risk predictions for intervening.^36^^,^^39^^,^^40^ While informative of construct validity, appropriateness does not represent a model’s usefulness over alternatives. If a mortality alert rule resulted in alerts for every hospitalization—and only hospitalizations—with a DNR in the CDSS-eligible cohort, its positive predictive value for 1-year mortality would be 43.5% (n = 2314 of 5325) and all cases would be appropriate for hypothetical CSO documentation; yet, this rule is useless for improving this outcome because it tells clinicians what they already act upon. A similar situation could result from using a model that is highly influenced by terms like “palliative” and “DNR,”^41^ or a model that uses historical palliative care consults to predict future consults.^37^ Even if alerts correctly predict mortality or benefit, those who would benefit from usual care anyway might be disproportionately identified. More concerningly, those who do not usually benefit may be further marginalized.^7^

Prediction models are often evaluated in biased conditions^42^ and rarely compared against routine clinical decision making.^43^ Clinical utility metrics—like an intention-to-treat estimator,^10^ the NB,^15^ or the NNB^16^—allow for patient-centered comparisons of prediction models with more appropriate assumptions. They can also detect unexpected differences in potential impact, like the difference in expected value between our 2 sites, before any health system investment and exposure to patients. We demonstrated 3 innovative strategies to increase the applicability of decision analysis for assessing the utility of automatable prediction models.

First, we did not rely on a link between risk threshold and clinical preference for net benefit analysis.^27^^,^^44^ Instead, we linked the risk threshold to the desired alert prevalence, representing resource use, and used other procedures to value outcomes. In doing so, we overcome a limitation of threshold-based NB analysis, which has been remarked as inappropriate for prediction model use cases that require considering resource availability in addition to patient benefits and harms.^16^ Note that the intent behind NB analysis—if not most decision-analytic methods^14^—is that it be adapted and extended to specific scenarios,^27^^,^^45^ the motivating principle being precisely that off-the-shelf metrics are not necessarily appropriate for all scenarios and stakeholders.^17^

Second, we extended the original decision tree used for decision curve analysis to allow simulating model-augmented outcomes (eg, that no alert can still lead to CSO if clinically appropriate), rather than model-determined outcomes (eg, that no alert will lead to no CSO). We would not want or expect the latter for our use case. By design, the adapted decision tree results in a more modest estimation of utility, one that accounts for the expected value of routine care: models can only increase benefit if it is there to be increased after applying usual clinical decision making.

Third, we used empiric rates of TP and FP actions to inform an observed exchange rate. This enabled decision curve analysis while comparing models at the same alert prevalence, which was not necessarily at the same risk threshold across models. Among those with a CSO, most patients preferred a DNR when the predicted risk of mortality was above 10% to 15%, but such a risk threshold would result in an alert prevalence over 50%. While likely acceptable for patients, who have little to lose and much to gain from a routine GOC discussion, this low risk threshold could imply unreasonable workloads for clinicians and cause alert fatigue.^12^ The observed exchange rate is a simple measure of the benefit-for-time trade-off that limits a good practice with minimal intervention-related harm. It is readily reproducible if practice patterns change over time and we believe it is insightful about clinical decision making, noting that physicians may be influenced by an inflated perception of GOC-related cost.^46^ This technique could facilitate the clinical utility assessment of other models for improving good practices in time-constrained environments, in which utilities cannot be inferred from the desired risk threshold. We used CSOs because they were the only electronic indicator of GOC documentation at our institution, but the same technique could be applied for other standardized indicators of the EOL communication process, like Physician Orders for Life-Sustaining Treatment.^47^

Our reproduction of the mHOMR model did not discriminate 1-year mortality as well as in Ontario (C-statistic 0.84 vs 0.89),^21^ but it was relatively simple to generalize to our institution. We cannot say the same of our ML model, which relies on admission diagnoses in Quebec-local French and would need another free-text mapping to be transportable beyond provincial borders (we report all variable definitions to enable this). However, while the local instance of our ML model is less geographically transportable than mHOMR, it is convincingly more useful for future application at our institution. This finding adds evidence to the recommendation that the pursuit of model generalizability should not be at the expense of local clinical utility.^13^

Our study has several limitations. First, resuscitation preference documentation is an essential but limited measure of EOL communication.^24^ We did not measure the quality of the GOC discussions that preceded a CSO, nor the concordance of preferences with care received.^5^ However, the role of this study was to inform implementation and not substitute a prospective evaluation of clinical impact, in which these higher-value patient outcomes should be assessed before long-term adoption.^8^ Second, decision analysis requires simplifying assumptions to be practical, like assuming that alerts would deterministically lead to action.^10^^,^^27^ To increase the transparency of these assumptions, we repeated analyses with a random model. In practice, some alert fatigue should be expected and addressed during pilot implementation (eg, by tailoring alerts to service needs).^12^ Third, owing to the COVID-19 pandemic, a repeat validation is likely warranted before local application because models rely on noncausal associations, such as between admission service and death, that may have unexpectedly shifted after systemic reorganization. Finally, although evaluating clinical utility of a prediction model is recommended and provides more value-based metrics than evaluating just predictive performance,^8–10^^,^^14–17^ more research is required to investigate how well these metrics predict the actual impact of a model-based CDSS. Future studies can refine on decision analysis based on this retroactive feedback, like including model-independent effects from behavioral economics–inspired cointerventions.^48^

## CONCLUSION

An evaluation of clinical utility, such as by using decision curve analysis, is recommended after validating a prediction model because metrics of model predictiveness, such as the C-statistic, are not informative of clinical value. This is particularly important for mortality prediction models having the use case of automatically prompting a PEOLC intervention, like a GOC discussion. Decision-analytic techniques to assess utility along patient-centered outcomes are feasible for quality improvement teams. They can help discriminate value from hype, calibrate expectations, and provide valuable information before CDSS implementation. As an adjunct to model validation, the routine evaluation of clinical utility could increase the value of automated predictive analytics implemented at the point of care.

## FUNDING

This study was supported by a clinician-investigator training grant (MR1-291226) funded jointly by the Fonds de Recherche du Quebec—Santé and the Ministère de la Santé et des Services Sociaux (to RT) and a clinician-investigator grant (CC-253453) from the Fonds de Recherche du Quebec—Santé (to J-FE). The Fonds de Recherche du Quebec—Santé and the Ministère de la Santé et des Services Sociaux had no role in the design and conduct of the study; collection, management, analysis, and interpretation of the data; preparation, review, or approval of the manuscript; and decision to submit the manuscript for publication .

## AUTHOR CONTRIBUTIONS

RT and J-FE were involved in conceptualization, funding acquisition, methodology, resources, validation, and writing (review and editing). RT was involved in data curation, formal analysis, software, visualization, and writing (original draft).

## SUPPLEMENTARY MATERIAL

Supplementary material is available at *Journal of the American Medical Informatics Association* online.

## Supplementary Material

ocab140_Supplementary_Data
